# Genomic Destabilization Triggered by Replication Stress during Senescence

**DOI:** 10.3390/cancers9110159

**Published:** 2017-11-21

**Authors:** Yusuke Minakawa, Atsuhiro Shimizu, Yusuke Matsuno, Ken-ichi Yoshioka

**Affiliations:** 1 Division of Carcinogenesis and Cancer Prevention, National Cancer Center Research Institute, 5-1-1 Tsukiji, Chuo-ku, Tokyo 104-0045, Japan; yminagaw@ncc.go.jp (Y.M.); atshimiz@ncc.go.jp (A.S.); yumatsun@ncc.go.jp (Y.M.); 2 Department of Biological Science and Technology, Tokyo University of Science, 6-3-1 Niijuku, Katsushika-ku, Tokyo 125-8585, Japan; 3 Department of Biosciences, School of Science, Kitasato University, 1-15-1 Kitasato, Minami-ku, Sagamihara 252-0373, Japan; 4 Department of Applied Chemistry, Tokyo University of Science, 1-3 Kagurazaka, Shinjuku-ku, Tokyo 162-8601, Japan

**Keywords:** genomic instability, senescence, DNA replication stress, cancer development

## Abstract

Most cancers develop after middle age, and are often associated with multiple mutations and genomic instability, implying that genomic destabilization is critical for age-related tumor development. In this manuscript, we review current knowledge regarding (1) the senescent cellular background, which is associated with a higher risk of genomic destabilization; and (2) the contributions of genomic destabilization to cancer development.

## 1. Introduction

Most cancers develop after middle age and are associated with various types of genomic instability, either chromosomal instability (CIN) or microsatellite instability (MSI) [1,2,3,4]. Cancer risk increases as a function of age [5,6]. Consistent with this, genomic destabilization is often observed in the senescent cellular state [7,8,9]. Such genomic instability is closely linked to cancer development. For example, mutations in the genes encoding breast cancer susceptibility 1 (BRCA1) and 2 (BRCA2) induce massive CIN due to a deficiency in homologous recombination, resulting in predisposition to cancer [10,11].

After serial proliferation, normal cells generally enter a growth-arrested state such as senescence or quiescence [12,13,14]. In quiescence, which is associated with low levels of H2AX, cells are protected from genomic destabilization [9,15,16]. Cells in this state are abundant in normal organs, and contribute to homeostasis [9]. By contrast, senescent cells accumulate persistent DNA double strand breaks (DSBs), and consequently contain numerous γH2AX foci [17,18]. This cellular state is associated with both aging-related diseases and precancerous states [18]. Senescence was long thought to be a mode of cancer prevention, partly because the tumor-suppressor protein p53 induces senescence when cells are damaged [19,20,21,22]. However, recent findings have revealed that senescence is often closely associated with genome destabilization and elevated cancer risk [23,24,25].

In this manuscript, we review the accumulated knowledge regarding the senescent cellular background associated with genomic destabilization, as well as its contributions to cancer development.

## 2. Genomic Instabilities in Cancer Cells and Cells Immortalized In Vitro

CIN and MSI are induced in cancer cells in a mutually exclusive manner, determined by mismatch repair (MMR) status (Table 1): MSI is induced in MMR-deficient backgrounds [26,27], whereas CIN is induced in most other cases [2,28]. MSI involves alterations in the lengths of microsatellite fragments, which contain short repetitive sequences (1–6 bases) [29]. CIN encompasses a wide variety of chromosomal abnormalities, including gene amplification [30], chromosomal deletions [31,32], chromosomal rearrangements [33], tetraploidy/aneuploidy [2], and loss of heterozygosity (LOH) [34] (Table 1). Recent work showed that CIN can also involve chromothripsis, i.e., multiple genomic rearrangements induced during a single catastrophic event, or chromoplexy, i.e., a complex DNA rearrangement caused by multiple DNA-strand breaks and their ligation into a new configuration [35,36]. Intriguingly, similar forms of genomic instability are also observed in cells immortalized or transformed in vitro [37,38]. Mouse embryonic fibroblast cells (MEFs) generally immortalize with destabilized genomes and mutations in the ARF/p53 module, whereas MEFs cultured under conditions that enable maintenance of genome stability never immortalize [9,39], suggesting that genomic instability abrogates the ARF/p53-dependent barrier.

## 3. Genomic Destabilization and Replication Stress

Genomic destabilization is generally caused by DSBs followed by erroneous repair [40,41]. It has been firmly established that ATM/ATR-mediated damage responses are massively activated in response to DSBs, leading to induction of repair or apoptosis, and thus serve as a barrier against genomic destabilization to prevent cancer [7,42,43]. In this context, a critical question is how such barrier systems are over-ridden during genomic destabilization. Several lines of evidence suggest that this phenomenon is associated with down-regulation of the variant histone H2AX. H2AX mediates efficient checkpoint responses and DSB repair [44,45], and is significantly down-regulated when the growth rate of normal cells decreases [9]. In fact, as shown in vivo and in vitro in both human and mouse cells, H2AX is down-regulated when normal cells enter a growth-arrested state. Although the mechanisms involved in down-regulation of H2AX in quiescent cells are not fully understood, it is clear at least that (1) H2AX down-regulation in undamaged cells is partly mediated by proteolytic degradation, mediated by the E3-ligase HUWE1 [46]; and (2) the formation of the H2AX-down-regulated state is dependent on both ARF and p53 and, hence, usually cannot occur after transformation and/or immortalization [9,15,39]. Unlike cells arrested in response to cell-cycle checkpoint signaling, cells in this state still progress through cell-cycle phases, especially under accelerated growth stimuli. Therefore, cells in this state are defective in repairing DSBs caused by replication stress, and are consequently vulnerable to genomic destabilization [47,48,49]. These issues are clearly illustrated in MEFs: MEFs grown under the 3T3 protocol maintain genome stability during primary growth, but become vulnerable to genomic destabilization when their growth rate decreases, even though the cultivation conditions are unchanged [39,50]. This risk of genomic destabilization is associated with replication stress; accordingly, genome stability is effectively maintained when exogenous growth stimuli are diminished [39,50]. Thus, normal cells generally become susceptible to genomic destabilization in the presence of high levels of growth stimuli.

**Figure 1:**
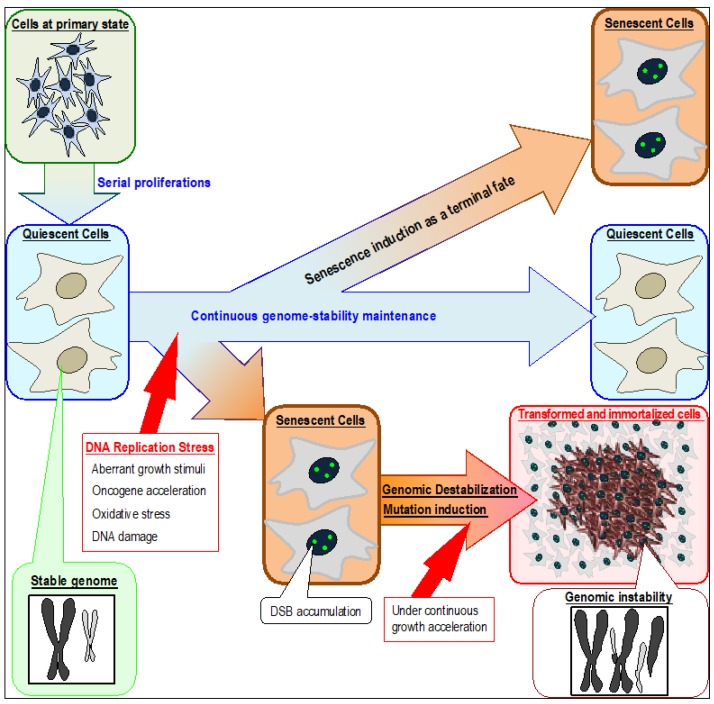
Induction of senescent and transformed cells triggered by DNA replication stress in association with genomic destabilization. Although quiescent cells can continuously preserve their cellular state and maintain genome stability, senescence can be induced by stresses that cause replication-fork stalling in association with persistent DSB (DNA double strand break) accumulation. Normal quiescent cells are defective in repairing DSBs caused by replication stress and are, therefore, vulnerable to genomic destabilization. Replication stress occurs in association with fork stalling, which can arise by multiple reasons that include aberrant growth stimuli, oncogene activation, oxidative stress, and DNA damage. The resultant cells accumulate DSBs and exhibit senescent characteristics. Immortalized and transformed cells usually appear after genomic destabilization.

Genomic destabilization commonly occurs when cells are subjected to replication stress (Figure 1), as is often observed in cells at precancerous stages [8,51,52,53]. By contrast, cells respond differently to DSBs directly caused by γ-rays [17,18,54], which are efficiently repaired through transient up-regulation of H2AX mediated by ATM and SIRT6 [46]. DSBs caused by replication stress do not induce H2AX expression and, therefore, persist [55]. The resultant cells usually accumulate a few γH2AX foci and express senescent characteristics [17,55]. Such cells still progress through the cell cycle in response to exogenous growth stimuli; consequently, DSBs are often carried over into mitosis, causing failures in cytokinesis and promoting the development of CIN with tetraploidy [37]. Since H2AX mediates checkpoint-response activation, such DSB carryover through the cell cycle is likely to be associated with down-regulation of H2AX. In support of this idea, the cellular H2AX level is associated with sensitivity to certain anti-cancer drugs: cancer cells treated with camptothecin accumulate H2AX and massively activate checkpoint responses, thereby effectively inducing apoptosis, whereas normal cells tend to survive in a H2AX-diminished state [50].

## 4. Genomic Destabilization and Induction of Cancer-Driver Mutations

How does genomic destabilization contribute to cancer development? Based on the knowledge accumulated to date, induction of mutations is the primary factor. In fact, as shown in colon, benign tumors mutated in Adenoma polyposis coli (APC) usually develop genomic instabilities [1,56]. The involvement of genomic destabilization in mutation induction is clearly illustrated in MEFs. Although immortalized MEFs, usually mutated in either ARF or p53, only appear after genomic destabilization, normal MEFs are prevented from immortalizing as long as genome stability is maintained in the quiescent state, which is established and maintained by both ARF and p53, concomitant with down-regulation of H2AX [9,39]. Thus, genomic destabilization might be a major cause of cancer-driver mutations.

Cancer risk is associated with the total number of stem cell divisions [57,58,59], which could be interpreted to mean that cancer risk rises in association with replication errors. However, this interpretation is unlikely to apply in multiple situations. In fact, cancers in colon invariably develop genomic instabilities (either CIN or MSI), except for cancer cells with hypermutation due to a proofreading-deficient mutation in Polε [60,61]. In addition, adenomas in colon often develop with tetraploidy, in which each gene is present in four copies, including the genes encoding APC, ARF, and p53 [62,63,64]. It is statistically impossible to stochastically mutate all four loci by simple replication errors; however, loss of all four copies could occur via genomic destabilization, e.g., through LOH. In this case, the elevated cancer risk in highly dividing stem cells could be due to the greater chance that replication stress will cause genomic destabilization, resulting in cancer-driving mutations.

As recently illustrated, hypermutation is often massively induced in association with kataegis, i.e., localized hypermutation in close proximity to genomic rearrangements [65]. This supports the aforementioned association between genomic destabilization and mutation induction, although the mechanism by which those mutations are induced remains elusive.

## 5. Oncogene Acceleration and Its Contribution to Cancer Development

Genomic destabilization is also caused by oncogene activation [7,8,37]. Oncogene-activated cells are widely subjected to accelerated S-phase entry because, as with exogenous growth stimuli, a major effect of oncogenes is cell-cycle progression [37,66]. Consequently, the resultant cells accumulate DSBs in association with replication stress, eventually express senescent characteristics [25,67], and are at higher risk of genomic destabilization [37].

Each of the four Yamanaka factors (c-Myc, Oct3/4, Sox2, and Klf4) was originally characterized as an oncogene [68,69,70]. However, in contrast to the effects of expressing any of them individually, simultaneous expression of all four factors leads to development of iPS cells [71]. This phenomenon implies that oncogene activation causes dedifferentiation effects, potentially contributing to the development of cancer stem cells (CSCs). Consistent with this idea, c-Myc, as well as oncogenic K-Ras, is widely used to achieve cellular transformation with tumorigenicity [72,73,74], a key characteristic of CSCs [75]. In fact, because immortalized MEFs do not exhibit CSC characteristics, they usually exhibit no or very limited tumor-formation ability; however, they can acquire tumor-formation ability after transformation with c-Myc, implying that this factor induces reprogramming and acquisition of CSC characteristics [67,76,77]. These lines of evidence suggest that c-Myc promotes cancer development via cellular reprograming, but the underlying mechanism remains unclear. In addition, it remains to be determined how c-Myc and K-Ras cause cellular transformation, unlike many other oncogenes, and how simultaneous expression of the four Yamanaka factors (as opposed to their individual expression) leads to cellular reprograming without causing replication stress and resultant genomic destabilization.

## 6. The Senescent Cellular Background and Its Contribution to Cancer Development

**Figure 2:**
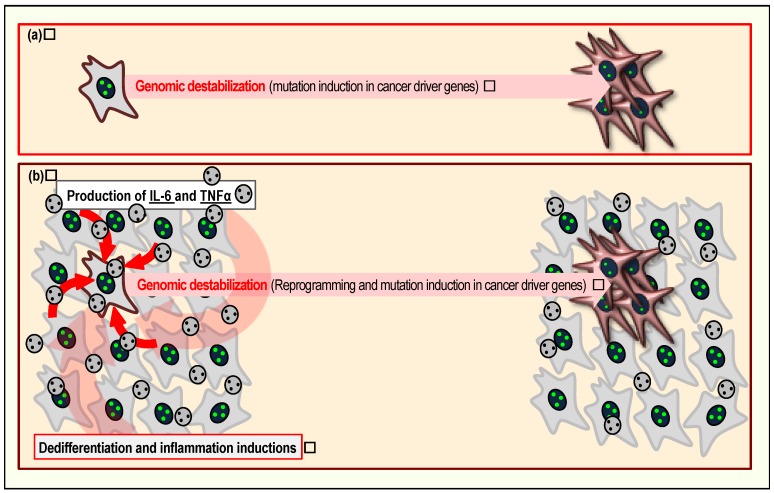
Induction of senescent cells promotes cancer development. Cells in the senescent state can contribute to cancer development in at least two ways. First, under continuous growth stimuli, accumulated DSBs can trigger genomic destabilization, directly promoting immortalization and acquisition of CSC (cancer-stem cell) characteristics, thereby promoting tumor development (**a**). Second, senescent cells often produce factors associated with the senescence-associated secretory phenotype, including TNFα and IL6 (**b**). Secreted TNFα and IL6 induce dedifferentiation and inflammation, further contributing to cancer development.

Genomic destabilization is generally triggered by erroneously repaired DSBs, and a large proportion of DSBs are associated with replication stress [40,41]. Consequently, cells in this state generally exhibit senescent phenotypes in response to accumulated DSBs [55] (Figure 2a). Cells containing accumulated DSBs that exhibit senescent phenotypes might be distinct from canonical senescent cells, notwithstanding the fact that DSBs accumulate in both types of cells. In fact, the canonical view is that senescence is induced as a form of cancer prevention, and represents a terminal fate [19,78]. However, multiple recent studies showed that cellular senescence can drive cancer development in some contexts [79]. In particular, DSB accumulation in cells with senescent phenotypes could trigger genomic destabilization, followed by immortalization and acquisition of CSC characteristics [38,80]. Since acquisition of such characteristics is dependent on cancer-driver mutations, such as loss of function in the ARF/p53 module, cells acquiring immortality or CSC characteristics must represent a minor fraction (Figure 2b); nonetheless, they inevitably appear, as demonstrated by multiple models including MEF immortalization [9,38,39].

Senescence with a higher risk of genomic destabilization is mainly caused by replication stress. In fact, cells at precancerous stages accumulate DSBs in association with replication stress and often exhibit senescent phenotype [8,45]. In addition, MEFs continuously cultivated under growth stimuli exhibit senescent phenotypes with DSB accumulation and develop genomic instability, and the resultant cells are subject to replication stress. By contrast, MEFs cultivated with reduced levels of growth stimuli are continuously quiescent and preserve their genome stability [9,39]. In support of these ideas, replication stress, genomic instability, and cellular senescence are simultaneously observed in cells exposed to aberrantly high levels of accelerated growth stimuli or oncogene activation [81]. Even in developed cancers harboring mutations in p53, a fraction of cells with senescent phenotypes is responsible for tumor progression [82]. Thus, senescent cellular states often drive cancer progression through genomic destabilization.

Senescent cells can promote cancer development in neighboring cells via multiple mechanisms. First, senescence in the stem cell niche can cause inappropriate stem cell differentiation, leading to CSC induction, as reviewed previously [16,83]. Second, Yamanaka factor expression in vivo induces senescence in some cells, which further contributes to reprogramming of neighboring cells via expression of IL6 and TNFα, leading to teratoma formation [84] (Figure 2b). Furthermore, IL6 and TNFα themselves mediate inflammation; therefore, cancer development can also be promoted in association with inflammation [85,86,87]. Specifically, cells subjected to inflammation express deaminase APOBEC3, thereby inducing deamination-mediated hypermutation [88,89]. Thus, the environmental effects caused by senescent cells contribute to an environment that promotes cancer. Among cells subjected to replication stress, those that develop immortality or CSC characteristics are dependent on mutations in cancer-driver genes and must, therefore, be a minor fraction; however, most of the surrounding cells still contribute to cancer progression via their environmental effects.

## 7. Age as a Risk Factor of Genomic Instability

Age is a risk factor for cancers that develop with genomic instability. Consistently, DSBs that risk genomic destabilization generally accumulate as a function of age [18]. Since cellular senescence is generally induced in response to DSBs [90,91,92] and is associated with organic aging [93,94], cells that accumulate DSBs are at a higher risk of cancer development through genomic destabilization. Although such DSB accumulation must be associated with a cellular state deficient in DSB repair, it remains unclear how cells in this state become repair-defective. This is partly because H2AX, which is essential for genome stability [45], is commonly down-regulated when normal cells enter a growth-arrested state [9]. However, this is probably not the only reason for the repair deficiency. In fact, senescent cells often form large γH2AX foci at DSB sites, even when the cellular H2AX level is low, but these lesions still do not undergo repair [18]. The mechanisms responsible for the repair defect remain to be elucidated, but this phenomenon might be due to dysfunction in sirtuin proteins SIRT1 and SIRT6. The sirtuin family regulates longevity and suppresses aging-related phenotypes [95,96]. In particular, SIRT1 and SIRT6 are localized in the nucleus, where they are involved in in multiple functions that include regulation of replication by SIRT1 [97] and mediation of DSB repair by SIRT6 [46,98,99].

Mammalian cells possess multiple types of barrier systems to defend themselves from cancer development. Based on recent knowledge, the risks of genome destabilization and the associated mutation are elevated when cells senesce. The induced mutations abrogate cancer-suppression systems, such as the ARF/p53 module.

## 8. Conclusions

Cancer is a disease associated with aging, and most cancers inevitably develop genomic instability. Based on recent knowledge, cellular senescence is tightly associated with cancer risk, primarily mediated by genomic destabilization, which is, in turn, associated with the induction of cancer-driver mutations. As cancer risk increases as a function of age, replication stress-associated DSBs that increase the risk of genomic destabilization also accumulate with age in vivo, causing cells to express senescent characteristics. Such DSB accumulation is induced in repair-deficient backgrounds partly because H2AX, which is required for recruitment of repair factors, is largely down-regulated when the growth rate of normal cells slows down. Consequently, the risk of genomic destabilization is generally elevated in such cellular backgrounds upon exposure to replication stress due to oxidation, aberrant growth stimuli, oncogene activation, or environmental factors that cause nucleotide adducts.

## Figures and Tables

**Table 1:** Types of genomic instability induced in cancer cells.

MMR Status	Type	Mutation Rate	Genomic Alteration	Description
Proficient	CIN	Low	Gene amplification	
			Chromosomal deletion	
			Chromosomal rearrangement	Frequently occurs at common fragile sites
			Tetraploidy/Aneuploidy	
			Loss of heterozygousity	
			Chromothripsis	Frequently occurs at common fragile sites
			Chromoplexy	
Deficient	MSI	High	Alteration in the lengths of microsatellite fragments	Frequently occurs at common fragile sites
